# A real-world cohort study of first-line afatinib in patients with EGFR-mutant advanced non-small cell lung cancer in Vietnam

**DOI:** 10.1186/s12885-024-11891-w

**Published:** 2024-02-05

**Authors:** Cam Phuong Pham, Thi Thai Hoa Nguyen, Anh Tu Do, Tuan Khoi Nguyen, Thi Anh Thu Hoang, Tuan Anh Le, Dinh Thy Hao Vuong, Dac Nhan Tam Nguyen, Van Khiem Dang, Thi Oanh Nguyen, Van Luan Pham, Minh Hai Nguyen, Thi Huyen Trang Vo, Hung Kien Do, Ha Thanh Vu, Thi Thuy Hang Nguyen, Van Thai Pham, Le Huy Trinh, Khac Dung Nguyen, Hoang Gia Nguyen, Cong Minh Truong, Tran Minh Chau Pham, Thi Bich Phuong Nguyen

**Affiliations:** 1 Bach Mai Hospital, Hanoi, Vietnam; 2 Vietnam National Cancer Hospital, Hanoi, Vietnam; 3 Ho Chi Minh City Oncology Hospital, Ho Chi Minh, Vietnam; 4 Cho Ray Hospital, Ho Chi Minh, Vietnam; 5 Thong Nhat Hospital, Ho Chi Minh, Ghana; 6 National Lung Hospital, Hanoi, Vietnam; 7 108 Military Central Hospital, Hanoi, Vietnam; 8 Hanoi Medical University, Hanoi, Vietnam; 9 Hanoi Oncology Hospital, Hanoi, Vietnam

**Keywords:** Advanced non-small cell lung cancer, EGFR mutations, Afatinib, First-line, Vietnam

## Abstract

**Background:**

This study aimed to evaluate the efficacy and side effects of first-line afatinib treatment in a real-world setting in Vietnam.

**Methods:**

This retrospective study was conducted across nine hospitals in Vietnam. Advanced epidermal growth factor receptor (EGFR)-mutant non-small cell lung cancer (NSCLC) patients who received afatinib as first-line therapy between April 2018 and June 2022 were included, and patient medical records were reviewed. Key outcomes were overall response rate (ORR), time-to-treatment failure (TTF), and tolerability.

**Results:**

A total of 343 patients on first-line afatinib were eligible for the study. EGFR exon 19 deletion (Del19) alone was detected in 46.9% of patients, L858R mutation alone in 26.3%, and other uncommon EGFR mutations, including compound mutations, in 26.8%. Patients with brain metastases at baseline were 25.4%. Patients who received 40 mg, 30 mg, and 20 mg as starting doses of afatinib were 58.6%, 39.9%, and 1.5%, respectively. The ORR was 78.1% in the overall population, 82.6% in the Del19 mutation subgroup, 73.3% in the L858R mutation subgroup, and 75.0% in the uncommon mutation subgroup (*p* > 0.05). The univariate and multivariate analyses indicate that the ORR increased when the starting dose was 40 mg compared to starting doses below 40 mg (83.9% vs. 74.3%, *p* = 0.034). The median TTF (mTTF) was 16.7 months (CI 95%: 14.8–18.5) in all patients, with a median follow-up time of 26.2 months. The mTTF was longer in patients in the common EGFR mutation subgroup (Del19/L858R) than in those in the uncommon mutation subgroup (17.5 vs. 13.8 months, *p* = 0.045) and in those without versus with brain metastases at baseline (17.5 vs. 15.1 months, *p* = 0.049). There were no significant differences in the mTTF between subgroups based on the starting dose of 40 mg and < 40 mg (16.7 vs. 16.9 months, *p* > 0.05). The most common treatment-related adverse events (any grade/grade ≥ 3) were diarrhea (55.4%/3.5%), rash (51.9%/3.2%), paronychia (35.3%/5.0%), and stomatitis (22.2%/1.2%).

**Conclusions:**

Afatinib demonstrated clinical effectiveness and good tolerability in Vietnamese EGFR-mutant NSCLC patients. In our real-world setting, administering a starting dose below 40 mg might result in a reduction in ORR; however, it might not have a significant impact on TTF.

**Supplementary Information:**

The online version contains supplementary material available at 10.1186/s12885-024-11891-w.

## Backgrounds

Lung cancer remains a significant public health concern worldwide, with its burden increasing in many countries, including Vietnam. In 2019, Vietnam was ranked 37th in terms of the lung cancer mortality rate globally [1]. According to Globocan 2020 statistics, there are approximately 26,262 new cases of lung cancer in Vietnam each year, accounting for 14.4% of all cancer cases and ranking second after liver cancer (14.5%) among different cancer types [2]. Lung cancer-related deaths comprised approximately 19% of total deaths in Vietnam, with lung cancer being the second leading cause of cancer-related deaths, accounting for 19.4% [2].

The number of new lung cancer cases is projected to continue rising in Vietnam, particularly in the two major cities of Hanoi and Ho Chi Minh City, due to population growth, aging, and the impact of smoking and secondhand smoke exposure [3]. The overall 5-year survival rate for lung cancer in Vietnam is 14.8% [1].

The prevalence of epidermal growth factor receptor (EGFR) mutations in Asian populations, including Vietnam, ranges from 39.6 to 51.4%, while specifically in Vietnam, the reported rate of EGFR gene mutations in patients with adenocarcinoma of the lung ranges from 40.7–64.2% [4, 5]. A recent study conducted in over 350 Vietnamese patients with non-small cell lung cancer (NSCLC) from four hospitals revealed the presence of EGFR mutations in 35% of cases, KRAS mutations in 23%, ALK rearrangements in 7%, ROS1 rearrangements in 3%, BRAF mutations in 2%, and NRAS mutations in 0.6% [6].

The current guidelines for the diagnosis and treatment of lung cancer in Vietnam recommend the use of first-generation (erlotinib, gefitinib), second-generation (afatinib), or third-generation (osimertinib) tyrosine kinase inhibitors (TKIs) as first-line treatment for advanced or metastatic NSCLC patients [7]. Afatinib, a second-generation TKI, has been approved for the treatment of EGFR-mutated NSCLC in Vietnam since 2018, with 50% coverage by health insurance [1, 7].

However, real-world data on the effectiveness and tolerability of afatinib in Vietnam are limited, as they have mostly been derived from single-center studies with small sample sizes [8].

Therefore, it is important to obtain representative real-world evidence on the effectiveness and tolerability of afatinib in NSCLC patients from different hospitals and cancer centers across Vietnam. This study aims to evaluate the response rate, time-to-treatment failure, and safety profile of first-line afatinib in EGFR-mutated NSCLC in a real-world multicenter setting in Vietnam.

## Patients and methods

### Study design

This multicenter retrospective observational study was conducted in nine hospitals across Vietnam. The nine hospitals are Bach Mai Hospital, Vietnam National Cancer Hospital, Ho Chi Minh City Oncology Hospital, Cho Ray Hospital, Thong Nhat Hospital, National Lung Hospital, 108 Military Central Hospital, Hanoi Medical University Hospital, and Hanoi Oncology Hospital.

EGFR mutation NSCLC patients who received afatinib as first-line treatment between April 2018 and June 2022 were enrolled, and patient medical records were reviewed. The study and protocol were approved by each Institutional Review Board. Key clinical outcomes included overall response rate (ORR), time-to-treatment failure (TTF), and tolerability. Key subgroup analyses included EGFR mutation categories, brain metastases at baseline, and starting dose/dose adjustment of afatinib.

### Study population

Patients were required to have histologically confirmed advanced NSCLC (inoperable IIIB-IIIC stage, recurrences, stage IV), first-line treatment, and EGFR mutations (including common mutations (Exon 19 deletion, L858R mutation) and other uncommon EGFR mutations). Patients with severe hepatic dysfunction (Child Pugh C) or renal impairment (estimated glomerular filtration rate < 15 mL/min/1.73 m2) or serious comorbidities, other malignant tumors, and the de novo T790M mutation were excluded.

### Procedures

EGFR mutations were detected in the pre-treatment biopsy specimens, mostly by PCR methods or next-generation sequencing. The initial afatinib dose, ranging from 20 mg to 40 mg, was selected by physicians’ decisions and the two most significant factors we considered in choosing the starting dose were age and Eastern Cooperative Oncology Group performance status (PS). Afatinib was given until symptomatic disease progression or the occurrence of intolerable side effects. In cases of limited progression, the decision to pursue definitive local therapy and to continue treatment with afatinib is made by a multidisciplinary team in the respective centers. The Response Evaluation Criteria in Solid Tumors 1.1 (RECIST 1.1) criteria were used to evaluate the best tumor response. The best clinical tumor response was recorded as complete response, partial response, stable disease, or progressive disease. The TTF is defined as the time from the first dose of afatinib to the date of treatment discontinuation due to progression, intolerance, or death. Patients who stopped treatment or switched to another EGFR TKI for other reasons were considered censored observations. Side effects documented in medical records were collected and graded according to the Common Terminology Criteria for Adverse Events (CTCAE 5.0) [9].

### Statistical analysis

Data were analyzed using IBM SPSS Statistics for Windows version 26.0 (IBM Corp., Armonk, NY). The presentation of continuous data as the mean ± standard deviation or median (interquartile range) and categorical data as the number (percentage) was dependent on their distribution. Differences between categorical variables were tested using the chi-square test or Fisher’s exact test. For continuous variables, the differences were compared using independent t tests or Mann‒Whitney U tests. Multivariable logistic binary regression with a forward selection approach was employed for the analysis of factors related to ORR. The TTF was estimated by the Kaplan–Meier method and compared by the log-rank test. A stepwise forward selection strategy was used to identify parameters for multivariate Cox proportional hazard regression models to evaluate factors associated with TTF. A *p* value of < 0.05 was considered statistically significant.

## Results

### Patient characteristics

There were initially 358 patients screened for first-line afatinib treatment and positive EGFR mutation. Following the exclusion of 15 patients based on the exclusion criteria, a total of 343 patients were included in the analysis. The patient characteristics grouped by starting dose of afatinib are described in detail in Table 1. A total of 137 patients received an initial dose of 40 mg afatinib, while 206 patients were in the group with an initial dose below 40 mg afatinib, predominantly at 30 mg, with only 5 patients starting at 20 mg. The average age of the patients in the study was 63.2 ± 9.7 years. Overall, 56.6% of patients were male, 93.1% had good performance status (PS 0–1), 72.6% were nonsmokers or former smokers, and 91.0% were in stage IV. Most patients had adenocarcinoma histology (98.0%), while a small percentage had squamous cell carcinoma (1.7%) or adenosquamous carcinoma (0.3%). The number of patients with brain metastases and liver metastases was 87 (25.4%) and 33 (9.6%), respectively. Common mutations, including exon 19 deletion (Del19) and L858R mutation (L858R), were observed in 251 patients (73.2%), with Del19 mutation in 161 patients (46.9%) and L858R mutation in 90 patients (26.2%). Details of uncommon mutation group were presented in Additional file 1. The patients who received < 40 mg daily as the initial dose tended to be older than those who received 40 mg daily (64.0 ± 9.6 vs. 61.9 ± 9.8 years); however, this difference was not statistically significant (*p* = 0.056). There were no significant differences in sex, smoking history, performance status, stage, brain metastasis, liver metastasis, or EGFR mutations (Del19/L858R/uncommon mutations) between the two groups of starting doses.

**Table 1:** Demographic and clinical characteristics of patients

Characteristic	All patients (*n* = 343)	Starting dose < 40 mg (*) (*n* = 206)	Starting dose 40 mg (**) (*n* = 137)	*p* value
**Age (years)**				
Mean ± SD	63.2 ± 9.7	64.0 ± 9.6	61.9 ± 9.8	0.056^a^
≥ 65 years old	152 (44.3)	98 (47.6)	54 (39.4)	0.136^b^
< 65 years old	191 (55.7)	108 (52.4)	83 (60.6)	
**Sex**				
Male	194 (56.6)	113 (54.9)	81 (59.1)	0.435^b^
Female	149 (43.4)	93 (45.1)	56 (40.9)	
**ECOG performance status at diagnosis**				
PS 0–1	319 (93.0)	193 (93.7)	126 (92.0)	0.541^b^
PS 2–3	24 (7.0)	13 (6.3)	11 (8.0)	
**Smoking history**				
Nonsmoker/former smoker	249 (72.6)	148 (71.8)	101 (73.7)	0.713^b^
Current smoker	94 (27.4)	58 (28.2)	36 (26.3)	
**Stage**				
IIIB/IIIC	19 (5.5)	7 (3.4)	5 (3.6)	0.955^b^
Recurrence	12 (3.5)	12 (5.8)	7 (5.1)	
IV	312 (91.0)	187 (90.8)	125 (91.2)	
**Sites of Metastasis**				
Brain	87 (25.4)	55 (26.7)	32 (23.4)	0.486^b^
Liver	33 (9.6)	18 (8.7)	15 (10.9)	0.496^b^
**EGFR mutations**				
Del 19	161 (46.9)	93 (45.1)	68 (49.6)	0.716^b^
L858R	90 (26.2)	56 (27.2)	34 (24.8)	
Uncommon mutations	92 (26.8)	57 (27.7)	25 (25.5)	

(*): 5 Patients starting dose 20 mg (**): no patients starting dose > 40 mg

a: T test b: Chi-square test

As shown in Table 2, most of the patients started with afatinib 30 mg once daily (58.6%), followed by 40 mg once daily (39.9%) and 20 mg once daily (1.5%). After one month of treatment, most of them could be maintained with the starting dose (81.9%). The number of patients requiring dose increases and dose reductions after one month was 25 (7.3%) and 37 (10.8%), respectively. Dose reductions due to tolerance were needed by 23.6% during the treatment. The optimal afatinib dosage, determined as the dose that could effectively manage the patient’s disease while maintaining tolerable side effects, was most prescribed at 30 mg once daily (62.1%), followed by 40 mg once daily (33.2%) and 20 mg once daily (4.7%). Among the patients with baseline brain metastases, 27.6% had concurrent whole brain radiation, and 16.1% had gamma knife radiosurgery.

**Table 2:** Afatinib dosage and treatment features

Characteristics	*N* = 343 (%)
**Starting dose**	
• 20 mg	5 (1.5)
• 30 mg	201 (58.6)
• 40 mg	137 (39.9)
**Dose adjustment after 1 month**	
• Dose increase	25 (7.3)
• Dose reduction	37 (10.8)
• Dose maintain	281 (81.9)
**Dose reductions during treatment**	
• Yes	81 (23.6)
• No	262 (76.4)
**Optimal dose**	
• 20 mg	16 (4.7)
• 30 mg	213 (62.1)
• 40 mg	114 (33.2)
**Treatment in the local of brain metastases (at the time of initiation of treatment)**	
• Whole Brain Radiation	24 (27.6)
• Gamma Knife	14 (16.1)
**Other palliative treatment**	
• Radiation relieves bone pain	10 (2.9)
• Radiation therapy to the chest	1 (0.3)
• Other	9 (2.6)

### Objective response

The overall objective response rate (ORR) was 78.1% in all patients, with 12.2% achieving a complete response and 65.9% achieving a partial response (Table 3).

**Table 3:** Overall Response Rate and Related Factors

Factors	ORR n (%)	Univariate analysis (*)		Multivariate analysis (**)		
		*p*	OR (95% CI)	*p*	OR (95% CI)	
**Best tumor response (n, %)**						
Complete response	42 (12.2)	*-*	***-***	***-***	***-***	
Partial response	226 (65.9)					
Stable disease	50 (14.6)					
Progressive disease	25 (7.3)					
**Age**						
• < 65 years old	117 (77.0)	0.643	1.00 (reference)	-	-	
• ≥ 65 years old	151 (79.1)		0.89 (0.53–1.48)			
**Gender**						
• Male	153 (78.9)	0.596	1.00 (reference)	-	-	
• Female	116 (77.8)		0.91 (0.54–1.52)			
**ECOG**						
• PS 0–1	253 (79.3)	0.055	1.00 (reference)	**0.049**	1.00 (reference)	
• PS 2–3	15 (62.5)		0.44 (0.18–1.04)		**0.41 (0.17–0.99)**	
**Smoking status**						
• Current smoker	73 (77.7)	0.896	1.00 (reference)	-	-	
• Non/former smoker	195 (78.3)		1.04 (0.59–1.84)			
**EGFR mutations**						
• Del 19	133 (82.6)	0.082^a^	1.00 (reference)	-	-	
• L858R	66 (73.3)		0.58 (0.31–1.08)			
• Del 19 + L858R	199 (79.3)	0.419^b^	-			
• Uncommon mutations	69 (75.0)		0.63 (0.34–1.18)			
**Stage**						
• IV	23 (74.2)	0.578	1.00 (reference)	-	-	
• IIIB, IIIC, recurrence	245 (78.5)		0.79 (0.34–1.84)			
**Brain metastasis**						
• Yes	62 (71.3)	0.073	1.00 (reference)	-	-	
• No	206 (80.5)		1.66 (0.95–2.90)			
**Starting dose**						
• 40 mg	115 (83.9)	**0.034**	1.00 (reference)	**0.029**	1.00 (reference)	
• < 40 mg	153 (74.3)		**0.55 (0.32–0.96)**		**0.54 (0.31–0.94)**	

(*): Chi square test

(**) Multivariable logistic binary regression (forward selection approach)

p^a^: L858R vs. Del 19

p^b^: Uncommon mutations vs. common mutations (Del19/L858R)

Among the common mutation group, the ORR for the Del 19 group was numerically higher than that for the L858R group; however, it is important to note that this difference did not reach statistical significance (82.6% vs. 73.3%, *p* = 0.082). The ORR for the uncommon mutation group (including compound mutations) was 75.0%, which tended to be lower than that of the common mutation group; however, this difference was not statistically significant (*p* = 0.419).

The ORR for the 87 patients with brain metastases was 71.3%, with 24 patients receiving whole-brain radiation therapy and 14 patients undergoing gamma knife radiosurgery (Table 2). This response rate was lower than that of the group without brain metastases (80.5%), but the difference was not statistically significant (*p* = 0.073).

Univariate analysis was performed for factors influencing the objective response rate, such as age (≥ 65 years, < 65 years old), sex, performance status (PS), smoking status, mutation type, disease stage, and brain metastases. However, no statistically significant differences were found in these factors (Table 3).

We utilized multivariable logistic binary regression with a forward selection approach to examine factors associated with ORR. Two factors were identified as related to ORR: performance status (PS 0–1 vs. PS 2–3) and starting dose (40 mg vs. <40 mg). (Table 3)

### Time to treatment failure

**Fig. 1:**
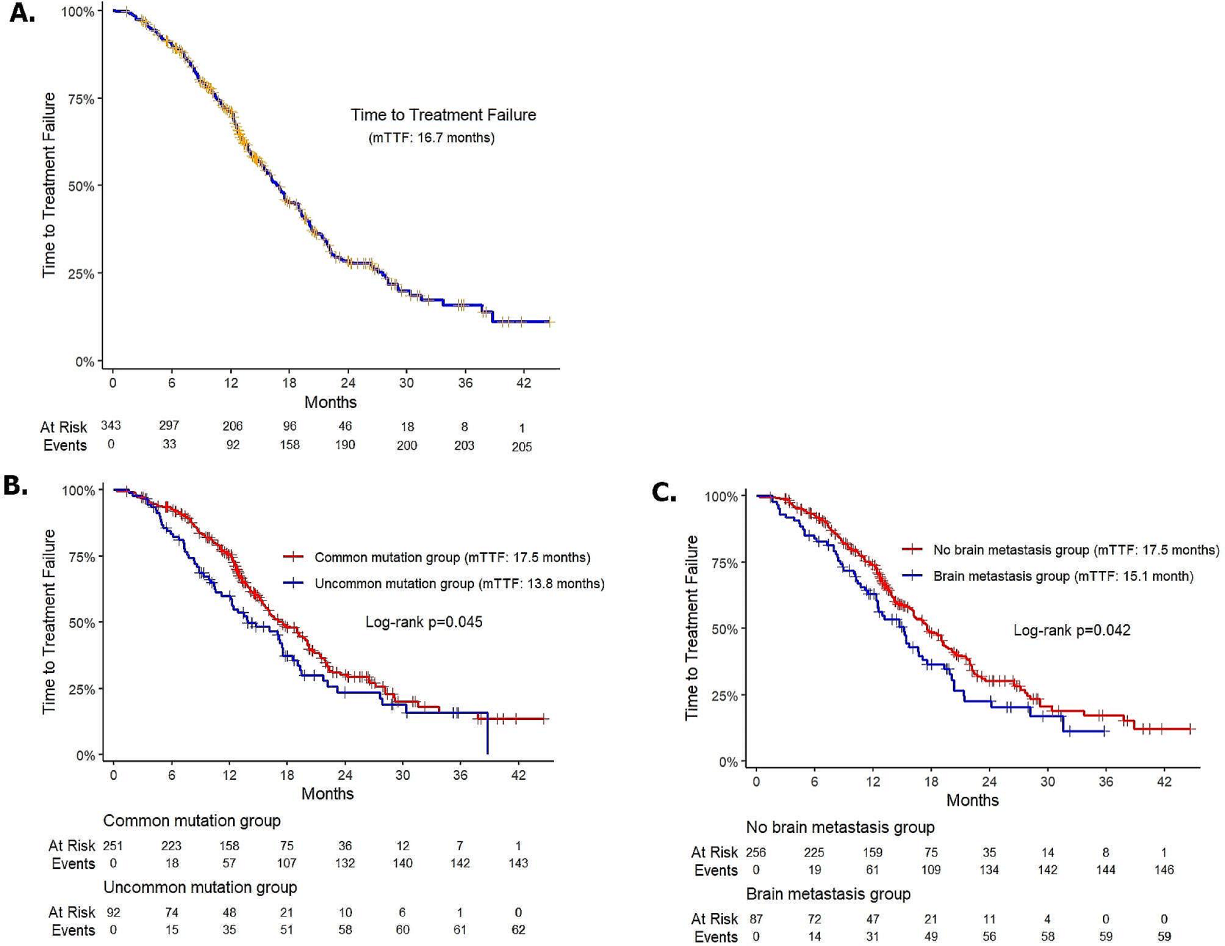
Kaplan–Meier curve of the TTF of the study population (**A**), the TTF stratified by EGFR mutations (**B**) and the TTF stratified by brain metastasis at baseline. Abbreviations: TTF: time-to-failure treatment, EGFR: Epidermal Growth Factor Receptor

The median follow-up time was 26.2 months ( interquartile range24.1 to 28.3 months) from the start of afatinib treatment. At the time of analysis in January 2023,OS data were immature, with 32.9% of events observed, while the median TTF was 16.7 months in all patients (Fig. 1a), with 59.8% of TTF events observed. Among the censored patients, 103 patients were still receiving afatinib, while 34 patients had to switch to another TKI due to financial problems or drug supply issues.

The mTTF was 17.5 months in the common mutation group and 13.8 months in the uncommon mutation group (*p* = 0.045) (Fig. 1b). In the group of patients with brain metastases, the mTTF was 15.1 months, which was significantly lower than that (17.5 months) in patients without brain metastases at baseline (*p* = 0.049) (Fig. 1c).

When analyzing the factors influencing TTF using Cox regression multivariate analysis, we observed that the mTTF was significantly lower in the uncommon mutation group (HR = 1.53, 95%CI: 1.13–2.07, *p* = 0.007) and the brain metastasis group (HR = 1.42, 95%CI: 1.04–1.92, *p* = 0.026)(Table 4).

**Table 4:** Time to Treatment Failure and Related Factors

Factors	mTTF (months)	Univariate analysis (*)		Multivariate analysis (**)	
		*p*	HR (95% CI)	*p*	HR (95% CI)
**Age**					
• < 65 years old	15.7	0.712	1.00 (reference)	-	-
• ≥ 65 years old	17.5		0.95 (0.72–1.25)		
**Gender**					
• Male	15.7	0.056	1.00 (reference)	-	-
• Female	19		0.76 (0.58–1.01)		
**ECOG**					
• PS 0–1	16.9	0.878	1.00 (reference)		
• PS 2–3	15.7		0.96 (0.57–1.63)		
**Smoking status**					
• Current smoker	17.5	0.243	1.00 (reference)	-	-
• Non/former smoker	15.4		0.83 (0.61–1.13)		
**EGFR mutations**					
• Del 19	17	0.225^a^	-	-	-
• L858R	19.6		-	-	-
• Common mutation	17.5	**0.045** ^**b**^	1.00 (reference)	**0.007** ^**b**^	1.00 (reference)
• Uncommon mutations	13.8		**1.36 (1.01–1.83)** ^**b**^		**1.53 (1.13–2.07)** ^**b**^
**Stage**					
• IV	16.7	0.666	1.00 (reference)	-	-
• IIIB, IIIC, recurrence	17.5		0.90 (0.55–1.47)		
**Brain metastasis**					
• No	17.5	**0.042**	1.00 (reference)	**0.026**	1.00 (reference)
• Yes	15.1		**1.37 (1.01–1.85)**		**1.42 (1.04–1.92)**
**Liver metastasis**					
• No	17	0.2	1.00 (reference)	-	-
• Yes	12.5		1.32 (0.86–2.03)		
**Starting dose**					
• 40 mg	16.7	0.755	1.00 (reference)	-	-
• < 40 mg	16.9		1.05 (0.79–1.38)		
**Optimal dose**					
• 40 mg	15.2	**0.003**	1.00 (reference)	**0.041**	1.00 (reference)
• < 40 mg	18.5		**0.65 (0.48–0.87)**		**0.72 (0.53–0.99)**
**Dose reduction**					
• No	15.7	**< 0.001**	1.00 (reference)	**0.003**	1.00 (reference)
• Yes	22		**0.54 (0.39–0.76)**		**0.58 (0.41–0.83)**

mTTF: median Time to Treatment Failure

(*): Log-rank test

(**) Cox regression multivariate analysis (forward selection approach)

p^a^: L858R vs. Del 19

p^b^: Ucommon mutations vs. common mutations (Del 19 + L858R)

### Dose adjustment

**Fig. 2:**
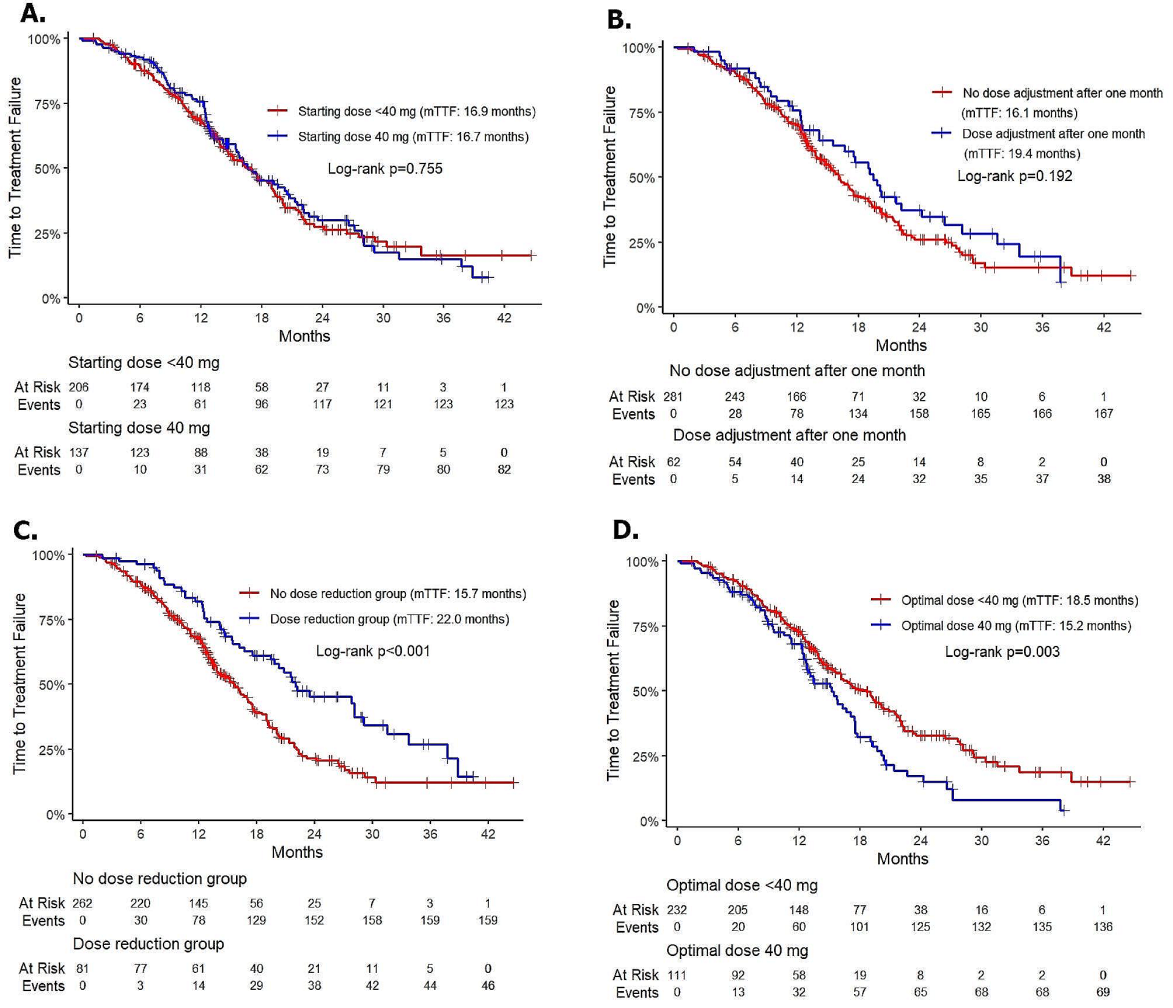
Kaplan–Meier curve of TTF among patients stratified by dosage factors: starting dose (**A**), dose reduction (**B**), dose adjustment after one month (**C**) and optimal dose (**D**). Abbreviations: TTF: time-to-failure treatment

The response rates in patients receiving an initial dose of 40 mg and < 40 mg were 83.9% and 74.3%, respectively, with a statistically significant difference (*p* = 0.034) (Table 3). On univariate analysis, the only factor found to have an influence on ORR was the starting dose, and it remained a prognostic factor in the multivariate analysis. However, there was no significant difference in mTTF between the two initial doses (16.7 months vs. 16.9 months, *p* = 0.755) (Fig. 2a).

The mTTF was significantly longer in patients with dose reduction than in those without dose reduction (22.0 months vs. 15.7 months, *p* < 0.001) (Fig. 2b). However, there was no significant difference in mTTF between the patients who needed dose adjustment, either through escalation or reduction, after one month of treatment and those who maintained their initial dose after one month (19.4 months vs. 16.1 months, *p* = 0.192) (Fig. 2c). Analyzing patients maintaining different optimal doses revealed that those with a tolerable dose of < 40 mg had a significantly longer mTTF than those with a tolerable dose of 40 mg (18.5 months vs. 15.2 months, *p* = 0.003) (Fig. 2d). Multivariate analysis indicated that dose reduction and optimal dose below 40 mg could result in bettermTTFs with HR = 0.58 (0.41–0.83) and HR = 0.72 (0.53–0.99), respectively (Table 4).

### Side effects

The most commonly observed adverse events included diarrhea (55.4%), rash (51.9%), paronychia (35.3%), stomatitis (22.2%), and dry skin (14.9%). Most of them were grades 1 and 2, and grade 3 was reported only with diarrhea (3.5%), rash (3.2%), paronychia (5.0%), and stomatitis (1.2%) (Table 5). No patients had grade 4 adverse events. The incidence of liver enzyme elevation was also low (9.9%), and no patients experienced interstitial lung disease. Diarrhea (any grade and grade 3) was more common in the group with a starting dose of 40 mg than in the group with a starting dose < 40 mg (*p* = 0.027 and *p* = 0.016, respectively). Stomatitis of any grade was more prevalent in the group with a starting dose of 40 mg (*p* = 0.047); however, there was no significant difference between the two groups in terms of grade 3 stomatitis.

**Table 5:** Most common treatment-related adverse events

	All patients ^a^	≤ 40 mg OD ^b, c^	40 mg OD ^c^	*p* value
(CTCAE grade)	*n* = 343 n (%)	*n* = 206 n (%)	*n* = 137 n(%)	
**Rash**				
• Any grade	178 (51.9)	110 (53.4)	68 (49.6)	0.495^d^
• ≥ G 3	11 (3.2)	5 (2.4)	6 (4.4)	0.315^d^
**Dry skin**				
• Any grade	51 (14.9)	27 (13.1)	24 (17.5)	0.261^d^
• ≥ G 3	0	0	0	-
**Paronychia**				
• Any grade	121 (35.3)	66 (32)	55 (40.1)	0.124^d^
• ≥ G 3	17 (5.0)	7 (3.4)	10 (7.3)	0.103^d^
**Diarrhea**				
• Any grade	190 (55.4)	104 (50.5)	86 (62.8)	**0.027** ^**d**^
• ≥ G 3	12 (3.5)	3 (1.5)	9 (6.6)	**0.016** ^**e**^
**Stomatitis**				
• Any grade	88 (22.2)	45 (21.8)	43 (31.4)	**0.047** ^**d**^
• ≥ G 3	4 (1.2)	1 (0.5)	3 (2.2)	0.306^e^
**GOT/GPT increase**				
• Any grade	28 (8.2)	16 (7.8)	12 (8.8)	0.742^d^
• ≥ G 3	0	0	0	-

OD: once daily

CTCAE: Common Terminology Criteria for Adverse Events

a: There was no grade 4 adverse event (%)

b: only 5 patients with a starting dose of 20 mg

c: Starting dose

d: Chi-square test

e: Fisher’s exact test

## Discussion

To the best of our knowledge, this is the largest real-world study to comprehensively evaluate the effectiveness of afatinib on the first-line treatment of NSCLC in Vietnam, which may provide valuable insights into patient characteristics and clinical outcomes. With the median follow-up duration of 26.2 months, the median TTF (mTTF) in overall population was 16.7 months. Compared to real-world data worldwide of mTTF ranging from 13.1 to 18.7 months [10–15], the Vietnamese population showed similar effectiveness of first-line afatinib. Additionally, in the study, patients with common mutation demonstrated significantly superior result relative to those harboring uncommon mutation subgroup, which generally consistent with reported data in the literature (Fig. 1b).

FLAURA study proved the superiority of osimertinib over first-generation EGFR TKI on progression free survival (PFS) and OS in NSCLC harboring common EGFR mutation with median PFS of 18.9 months. However, osimertinib failed to show benefit of OS in Asian patients. In our study, common mutation patients treated with afatinib showed promising outcome with mTTF of 17.5 months. These data support the advantage of sequential afatinib therapy followed by osimertinib in the Asian population, as echoed by results of some other real-word studies [10, 12, 16].

Patients with brain metastases baseline revealed significantly inferior treatment outcome relative to those without brain involvement, as predicted for this poor prognosis subgroup (Fig. 1c). Nevertheless, with the mTTF of 15.1 months, the effectiveness of afatinib in these patients was highly encouraging.Similar results were reported in studies from Korea (14.8 months) [10] and China (15.6 months) [17]. These findings support the clinical activity of afatinib in EGFR mutation-positive patients with NSCLC and asymptomatic brain metastases.

In a previous study in Vietnam, we observed that most patients were prescribed a starting dose of 30 mg of afatinib [8]. In this retrospective study, a significant proportion of patients began treatment with afatinib at 30 mg once daily (58.6%), followed by 40 mg once daily (39.9%) and 20 mg once daily (1.5%). This is different from other real-world studies in first-line afatinib in the region. The response rate for the 40 mg starting dose is significantly higher than that for the group below 40 mg (Table 3). In multivariate analysis regarding factors related to ORR, the starting dose 40 mg versus below 40 mg remained a prognostic factor for a better ORR. Studies with Taiwanese patients with EGFR Del 19 or L858R mutations showed no difference in the overall response rate between the 30 mg and 40 mg starting doses [18, 19]. The difference observed in our study may be attributed to the fact that more than 1/4 of the study population had uncommon mutations (26.8%). However, the difference between the initial doses did not affect the mTTF (16.7 months vs. 16.9 months, *p* = 0.755) (Fig. 2a). This is consistent with the findings of other real-world studies [18, 20–23].

As demonstrated in LUX-Lung program and RealGiDo study [14, 24, 25], tolerability guided dose adjustment improved the safety profile but not compromised the efficacy of afatinib. To investigate the generalization of the finding in Vietnamese population, the impact of dose adjustment was thoroughly evaluated in our study. The mTTF was significantly longer in patients who experienced tolerability guided dose adjustment than those who did not. Similarly, patients with the optimal dose of < 40 mg showed superior treatment outcome to those having 40 mg as the optimal dose. Our data echoes the finding that dose adjustment of afatinib help alleviate the frequency and severity of treatment-related adverse events (TRAEs) without negatively impacting clinical benefits [18, 22, 26].

Regarding adverse effects, we did not encounter any additional adverse effects other than those already documented and reported for afatinib (Table 5). Diarrhea (any grade and grade 3) was more common in the group with a starting dose of 40 mg than in the group with a starting dose < 40 mg. Conversely, stomatitis of any grade but not grade 3 or above was more prevalent in the subgroup with starting dose of 40 mg. The lower frequency observed in our study compared to previous prospective studies [24, 25] but comparable to real-world data in China [17] and South Korea [27] may be attributed to the nature of retrospective study designs.

The important limitation of the study was retrospective nature. Additionally, the multivariate analysis with a forward selection approach can be susceptible to selection bias. Furthermore, the decision of selecting the starting dose of afatinib varied by physicians according to performance status, co-morbidity, and clinical experience, challenging the interpretation of findings. Last, OS remained immature at the point of data cut-off for this analysis due to short follow-up. Despite these limitations, the study is the first multi-center study in Vietnam, representing real-world clinical practice and providing useful insights regarding the use of first-line afatinib for the treatment of advanced EGFR mutation-positive NSCLC. Our direction for future research is to conduct a prospective study to assess the efficacy of an initial 30 mg dose of afatinib in treating Vietnamese patients.

## Conclusion

This first multicenter real-world data from Vietnam demonstrate consistent effectiveness and tolerability of first-line afatinib in Vietnamese patients, aligning with randomized controlled trials (RCTs) and real-world evidence (RWE).

In summary, the multicenter real-world data from Vietnam confirm the effectiveness and tolerability of first-line afatinib in Vietnamese patients, consistent with RCTs and RWE. The study provides valuable insights into response rates, mTTF, and safety profiles, highlighting the importance of individualized dosing and proactive management of adverse events to optimize treatment outcomes. Further research and studies are warranted to enhance our understanding of afatinib in the Vietnamese population and improve patient care.

### Electronic supplementary material

Below is the link to the electronic supplementary material.

Supplementary Material 1

## Data Availability

The de-linked and anonymized datasets used and/or analyzed during the current study are available from the corresponding author on reasonable request.

## Abbreviations

CI: Confidence interval
CTCAE v5.0: Common Terminology Criteria for Adverse Events version 5
ECOG: Eastern Cooperative Oncology Group
EGFR: Epidermal growth factor receptor
HR: Hazard ratio
mTTF: Median time-to-treatment failure
NSCLC: Non-small cell lung cancer
OR: Odds ratio
ORR: Objective response rate
PS: Performance status
RCT: Randomized controlled trial
RWE: Real-World Evidence
SD: Standard deviation
TKI: Tyrosine kinase inhibitor
